# Impact of stress hyperglycemia ratio on mortality in patients with cardiac arrest: insight from American MIMIC-IV database

**DOI:** 10.3389/fendo.2024.1383993

**Published:** 2024-05-21

**Authors:** Li-You Lian, Wei-Hao Xue, Jia-Jia Lu, Ru-Jie Zheng

**Affiliations:** ^1^ Department of Cardiology, the First Affiliated Hospital of Wenzhou Medical University, Wenzhou, China; ^2^ Department of Cardiology, The Second Affiliated Hospital and Yuying Children’s Hospital of Wenzhou Medical University, Wenzhou, China; ^3^ Department of Public Education, Zhangzhou Institute of Technology, Zhangzhou, China; ^4^ Department of Radiology, the First Affiliated Hospital of Wenzhou Medical University, Wenzhou, China

**Keywords:** cardiac arrest, stress hyperglycemia ratio, diabetes, mortality, prognosis

## Abstract

**Background:**

Stress hyperglycemia ratio (SHR) has shown a predominant correlation with transient adverse events in critically ill patients. However, there remains a gap in comprehensive research regarding the association between SHR and mortality among patients experiencing cardiac arrest and admitted to the intensive care unit (ICU).

**Methods:**

A total of 535 patients with their initial ICU admission suffered cardiac arrest, according to the American Medical Information Mart for Intensive Care (MIMIC)-IV database. Patients were stratified into four categories based on quantiles of SHR. Multivariable Cox regression models were used to evaluate the association SHR and mortality. The association between SHR and mortality was assessed using multivariable Cox regression models. Subgroup analyses were conducted to determine whether SHR influenced ICU, 1-year, and long-term all-cause mortality in subgroups stratified according to diabetes status.

**Results:**

Patients with higher SHR, when compared to the reference quartile 1 group, exhibited a greater risk of ICU mortality (adjusted hazard ratio [aHR] = 3.029; 95% CI: 1.802-5.090), 1-year mortality (aHR = 3.057; 95% CI: 1.885-4.958), and long-term mortality (aHR = 3.183; 95% CI: 2.020-5.015). This association was particularly noteworthy among patients without diabetes, as indicated by subgroup analysis.

**Conclusion:**

Elevated SHR was notably associated with heightened risks of ICU, 1-year, and long-term all-cause mortality among cardiac arrest patients. These findings underscore the importance of considering SHR as a potential prognostic factor in the critical care management of cardiac arrest patients, warranting further investigation and clinical attention.

## Introduction

Approximately 420,000 individuals experience out-of-hospital cardiac arrest annually in the United States, with respiratory failure being the predominant cause in most cases (1, 2). Unfortunately, only 25% of these cases result in survival and discharge from the hospital. Even with a successful return of spontaneous circulation following out-of-hospital cardiac arrest, approximately 80% of patients remain comatose (3). Notably, cardiac arrest-associated central nervous system damage stands out as the primary cause of long-term disability and mortality among survivors of the acute resuscitation phase (4, 5). The persistent risk of ischemia/reperfusion injury affecting various organs continues to be a significant concern, impacting patient outcomes (1, 6–8). The timely administration of cardiopulmonary resuscitation (CPR) and defibrillation has been proven crucial for maintaining sustained autonomic circulation and improving survival rates. Consequently, there is an urgent need to identify effective and straightforward indicators that can accurately predict the prognosis of patients who have undergone cardiac arrest.

Stress hyperglycemia, a transient metabolic response characterized by elevated glucose levels in emergency situations, had been reported in studies involving patients in critical conditions (9, 10). This phenomenon had been associated with instability, perforation, and exacerbation of myocardial ischemia in atherosclerotic plaques, which are among the leading causes of cardiac arrest (11, 12). Despite its significance, there is currently no universally accepted definition of stress hyperglycemia (13). Patients with this condition are typically classified as having known diabetes, newly diagnosed diabetes, or hyperglycemia related to the hospital environment (14). In previous research, fasting glucose or initial blood glucose levels upon admission were commonly used as indicators of stress hyperglycemia. However, these measures often failed to consider the baseline glucose levels preceding the onset of cardiovascular-related diseases, particularly in emergency situations such as cardiac arrest (15, 16). Introducing the stress hyperglycemia ratio (SHR), a novel indicator calculated by dividing the measurement of admission blood glucose (ABG) by HbA1c, offered a more comprehensive assessment (17–19). Studies have indicated a strong association between SHR and adverse clinical outcomes in critically ill patients (20, 21). Nonetheless, limited research has explored the relationship between SHR and mortality in patients experiencing cardiac arrest.

Accordingly, the purpose of the present investigation was to examine the association between SHR and ICU, 1-year and long-term all-cause mortality in American Medical Information Mart for Intensive Care IV (MIMIC-IV) cohort cardiac arrest patients admitted to the intensive care unit (ICU).

## Methods

### Sources of data

This retrospective study utilized data from the MIMIC-IV database (22), established and maintained by the Massachusetts Institute of Technology’s Laboratory for Computational Physiology (23). The MIMIC database is an extensive, one-stop, publicly accessible database comprising data about patients admitted to critical care sections at a major tertiary care facility (23). Since 2008, 76,540 intensive care unit (ICU) patients have been entered into the Beth Israel Deaconess Medical Center in Boston database. Access to the database was granted to one of our group’s authors (Record ID: 5,076,556) upon successful completion of the Collaborative Institutional Training Initiative (CITI) program course. The data extraction process was executed utilizing PostgreSQL version 6.11.1 software.

**Figure 1:**
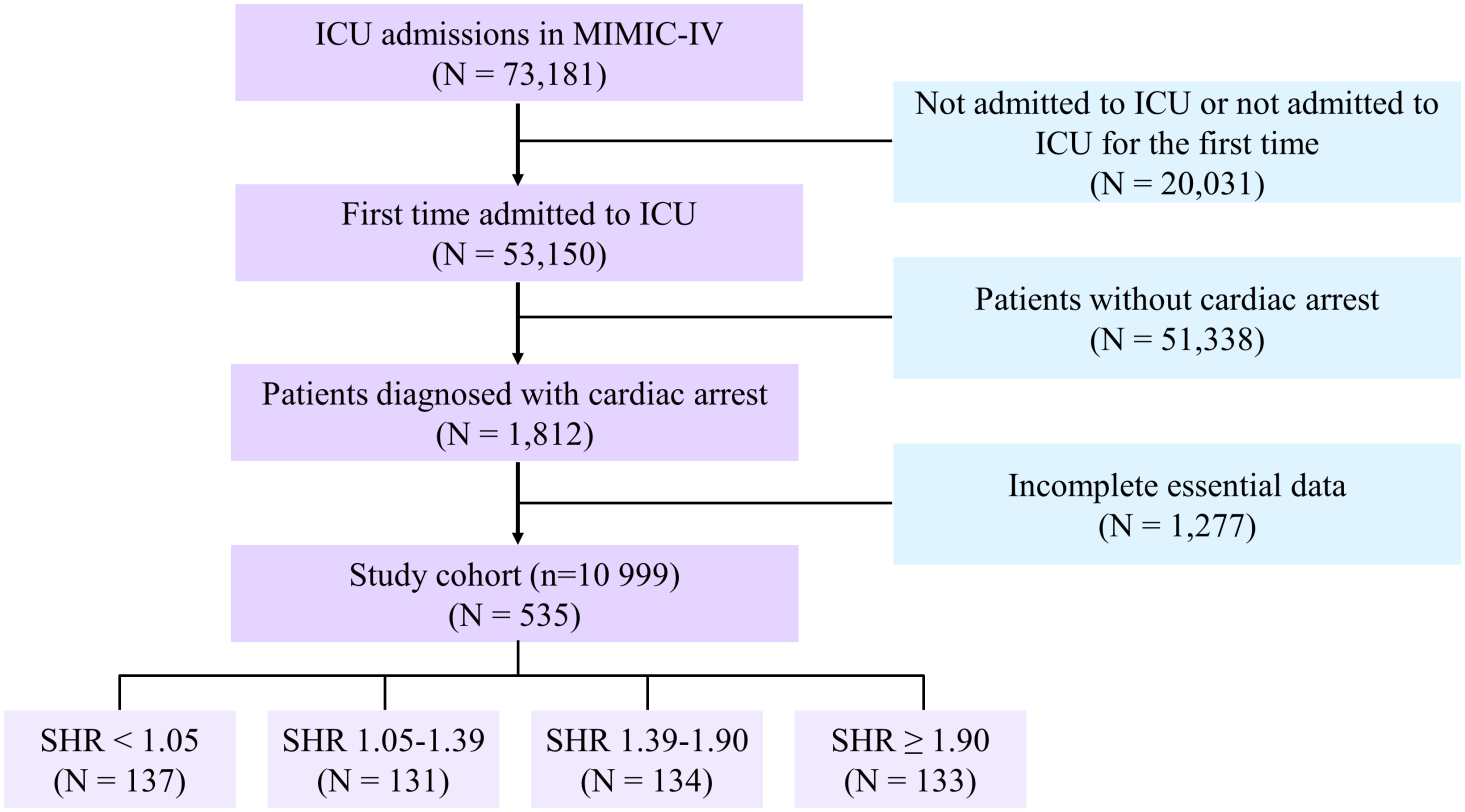
Study flowchart. SHR, stress hyperglycemia ratio; ICU, intensive care unit.

We obtained clinical data from ICU-admitted adults older than 18 years for the first time for the purpose of analysis. The individuals who were found to have cardiac arrest, as classified by the International Classification of Diseases (ICDS) 9th and 10th editions (ICD-9 codes “4275”, “I46”, “I462”, “I468”, “I469”), satisfied the requirements for this study. As screening criteria, the following exclusion criteria were applied: (1) patients younger than 18 years of age; (2) patients whose survival outcome data were unavailable; (3) insufficient or absent critical laboratory findings (glycated hemoglobin [HbA1c] or glucose on admission). All laboratory variables and disease severity scores were extracted from the data generated within the first 24 hours after the patient entered the ICU. Finally, 535 cardiac arrest patients were included in American MIMIC-IV (**Figure 1**).

### Outcomes and definitions

The study’s outcomes were ICU mortality, 1-year mortality and long-term mortality, with the MIMIC-IV cohort having a maximum follow-up of 12.1 years. The SHR index was established using the following formula: SHR = (admission glucose) (mmol/L) divided by (1.59 * HbA1c [%] - 2.59).

### Statistical analysis

When analyzing normally distributed values, we represented continuous data using the mean (standard deviation). For non-normally distributed values, we used the median (interquartile range). Categorical data was displayed as quantities and percentages. In order to compare the groups, appropriate statistical tests such as ANOVA, the Kruskal-Wallis test for continuous variables, or the chi-square test for categorical data were utilized. SHR was assessed as a categorical variable: quartiles 1 to 4. To calculate the cumulative hazard of mortality from all causes, the Kaplan–Meier method was applied. In order to ascertain the correlation between SHR level and ICU mortality, 1-year mortality, and long-term mortality, multivariable Cox regression models were employed. These models accounted for age, gender, hypertension, diabetes mellitus, chronic kidney disease, acute myocardial infarction (AMI), cerebrovascular disease, peptic ulcer disease, and chronic obstructive pulmonary disease (COPD). The correlation between SHR and ICU mortality, 1-year mortality, and long-term mortality was also examined using restricted cubic splines (RCS). In presenting the findings, hazard ratios (HR) and confidence intervals (CI) of 95% are employed. In addition, subgroup analyses were performed to ascertain whether SHR influenced mortality in intensive care units, mortality at one year, and mortality over the long term in subgroups stratified according to diabetes status. The statistical analysis was performed utilizing the R software (version 4.2.1). P values with both tails containing less than 0.05 were considered statistically significant.

## Results

The MIMIC-IV cohort enrolled 535 cardiac arrest patients (mean age [SD]: 66.9 ± 14.4 years; 62.6% male). In the MIMIC-IV cohort, patients were categorized into four groups according to their SHR level: quartile 1 [N =137], SHR<1.05; quartile 2 [N =131], 1.05≤SHR<1.39; quartile 3 [N =134], 1.39≤SHR<1.90; and quartile 4 [N =133], SHR≥1.90).

A total of 65.2% (N =349) of the patients in the MIMIC-IV cohort were diagnosed with hypertension, 56.4% (N =302) with diabetes, 60.9% (N =326) with chronic heart failure (CHF), 27.5% (N= 147) with AMI, and 16.3% (N =87) with CKD. Further elaboration on the foundational data of these groups could be found in **Table 1**.

**Table 1:** Baseline Characteristics of the Subjects.

Characteristics	Quartile 1	Quartile 2	Quartile 3	Quartile 4	P-value
	<1.05	1.05-1.39	1.39-1.90	>1.90	
	N=137	N=131	N=134	N=133	
SHR	0.83 (0.17)	1.21 (0.10)	1.61 (0.14)	2.83 (0.99)	<0.001
Age, years old	69.51 (14.43)	66.69 (16.27)	67.06 (12.69)	64.14 (13.90)	0.024
Gender, N (%)					0.931
Female	50 (36.50)	47 (35.88)	53 (39.55)	50 (37.59)	
Male	87 (63.50)	84 (64.12)	81 (60.45)	83 (62.41)	
Medical history, N (%)					
Acute Myocardial Infarct	33 (24.09)	43 (32.82)	43 (32.09)	49 (36.84)	0.146
Congestive Heart Failure	58 (42.34)	64 (48.85)	65 (48.51)	58 (43.61)	0.613
Peripheral Vascular Disease	33 (24.09)	19 (14.50)	24 (17.91)	17 (12.78)	0.071
Cerebrovascular Disease	22 (16.06)	29 (22.14)	26 (19.40)	23 (17.29)	0.601
Dementia	2 (1.46)	3 (2.29)	2 (1.49)	3 (2.26)	0.927
Chronic Pulmonary Disease	27 (19.71)	32 (24.43)	32 (23.88)	40 (30.08)	0.265
Rheumatic Disease	5 (3.65)	7 (5.34)	2 (1.49)	2 (1.50)	0.191
Peptic Ulcer Disease	3 (2.19)	2 (1.53)	7 (5.22)	0 (0.00)	0.032
Mild Liver Disease	9 (6.57)	15 (11.45)	13 (9.70)	10 (7.52)	0.492
Diabetes	87 (63.50)	60 (45.80)	72 (53.73)	83 (62.41)	0.011
Paraplegia	7 (5.11)	1 (0.76)	7 (5.22)	3 (2.26)	0.115
Renal Disease	45 (32.85)	42 (32.06)	47 (35.07)	44 (33.08)	0.961
Malignant Cancer	13 (9.49)	11 (8.40)	10 (7.46)	9 (6.77)	0.858
Severe Liver Disease	0 (0.00)	1 (0.76)	5 (3.73)	4 (3.01)	0.073
Metastatic Solid Tumor	3 (2.19)	5 (3.82)	4 (2.99)	2 (1.50)	0.669
COPD	15 (10.95)	6 (4.58)	10 (7.46)	19 (14.29)	0.04
HIV	0 (0.00)	1 (0.76)	3 (2.24)	2 (1.50)	0.335
Laboratory indexes					
Hemoglobin, g/dL	11.35 (2.00)	11.89 (2.34)	11.77 (2.20)	12.51 (2.57)	<0.001
Platelets, K/uL	219.32 (89.27)	218.98 (82.71)	244.95 (106.36)	259.47 (109.07)	<0.001
WBC, K/uL	12.53 (6.42)	14.05 (15.93)	15.69 (8.41)	19.87 (8.71)	<0.001
Albumin, g/dL	3.24 (0.67)	3.32 (0.68)	3.44 (0.61)	3.46 (0.63)	0.221
Anion gap, mEq/L	15.78 (4.12)	16.98 (4.63)	19.18 (4.63)	22.56 (5.54)	<0.001
Bicarbonate, mEq/L	24.88 (4.16)	24.66 (4.41)	24.28 (4.51)	22.42 (4.51)	<0.001
BUN, mg/dL	24.00 (17.00-39.00)	25.00 (17.50-40.50)	28.00 (20.00-47.75)	32.00 (22.00-51.00)	0.005
Calcium, mg/dL	8.74 (0.87)	8.69 (0.88)	8.92 (1.03)	8.96 (1.43)	0.135
Chloride, mEq/L	105.29 (5.45)	105.34 (6.36)	105.28 (7.64)	106.26 (6.38)	0.542
Creatinine, mg/dL	1.20 (0.90-1.80)	1.20 (1.00-2.00)	1.50 (1.10-2.35)	1.90 (1.20-2.90)	0.014
Glucose, mmol/L	7.25 (2.38)	9.43 (3.54)	12.52 (4.19)	22.74 (10.10)	<0.001
HbA1c, %	7.32 (2.04)	6.54 (1.83)	6.53 (1.56)	6.73 (1.81)	<0.001
Sodium, mEq/L	139.18 (3.97)	139.98 (4.79)	140.50 (5.91)	140.63 (5.07)	0.068
Potassium, mEq/L	4.56 (0.68)	4.84 (1.06)	4.85 (0.93)	5.24 (1.09)	<0.001
INR	1.40 (1.20-1.70)	1.40 (1.20-1.80)	11.30 (1.20-1.78)	1.40 (1.17-1.70)	0.697
PTT	35.50 (29.50-54.00)	35.90 (29.80-51.70)	46.70 (32.20-97.20)	54.00 (31.85-101.65)	<0.001
ALT, U/L	30.50 (17.00-63.00)	43.00 (23.75-87.50)	50.00 (24.75-126.75)	89.00 (44.00-233.00)	0.241
ALP, U/L	87.00 (64.50-133.00)	81.00 (62.50-118.50)	88.00 (64.50-135.00)	88.00 (64.50-122.00)	0.121
AST, U/L	41.00 (24.75-106.25)	60.00 (34.00-170.00)	75.00 (34.75-199.75)	171.00 (63.00-464.00)	0.749
Total bilirubin, mg/dL	0.60 (0.40-1.25)	0.70 (0.50-1.30)	0.70 (0.40-1.00)	0.60 (0.40-0.92)	0.643
Sofa score	5.00 (2.00-8.00)	5.00 (3.00-10.00)	7.00 (4.00-9.00)	9.00 (6.00-12.00)	<0.001

SHR, stress hyperglycemia ratio; COPD, chronic obstructive pulmonary disease; HIV, human immunodeficiency virus; INR, international normalized ratio; PTT, partial thromboplastin time; ALT, alanine transaminase; ALP, alkaline phosphatase; AST, aspartate transaminase; HbA1c, glycated hemoglobin.

**Figure 2:**
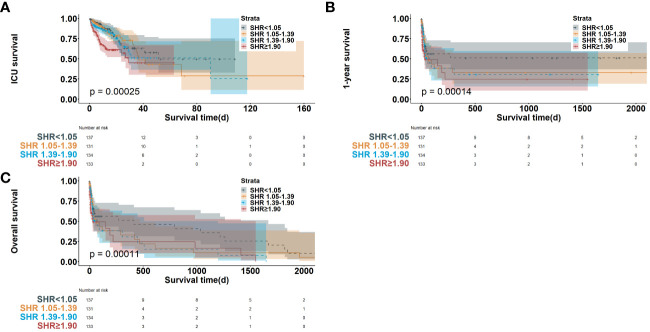
Kaplan-Meier survival curve of survival probability in cardiac arrest patients in ICU mortality **(A)**, 1-year mortality **(B)** and long-term mortality **(C)**. SHR, stress hyperglycemia ratio; ICU, intensive care unit.

All-cause mortality was observed in 169 patients (31.6%) of the American MIMIC-IV cohort; the greatest mortality rate was observed in quartile 4 (N = 53, 39.8%). The higher SHR was associated with higher mortality. The outcomes of Kaplan–Meier analyses of survival were illustrated in **Figure 2**.

The results of the RCS analyses revealed an L-shaped association between the SHR and all-cause mortality during the follow-up period (P value for nonlinearity >0.05 for all, **Supplementary Figure 1**). In the MIMIC-IV cohort, the SHR corresponding to the lowest risk on RCS was 1.39 of ICU mortality, 1-year mortality, and long-term mortality (**Supplementary Figure 1**).

**Figure 3:**
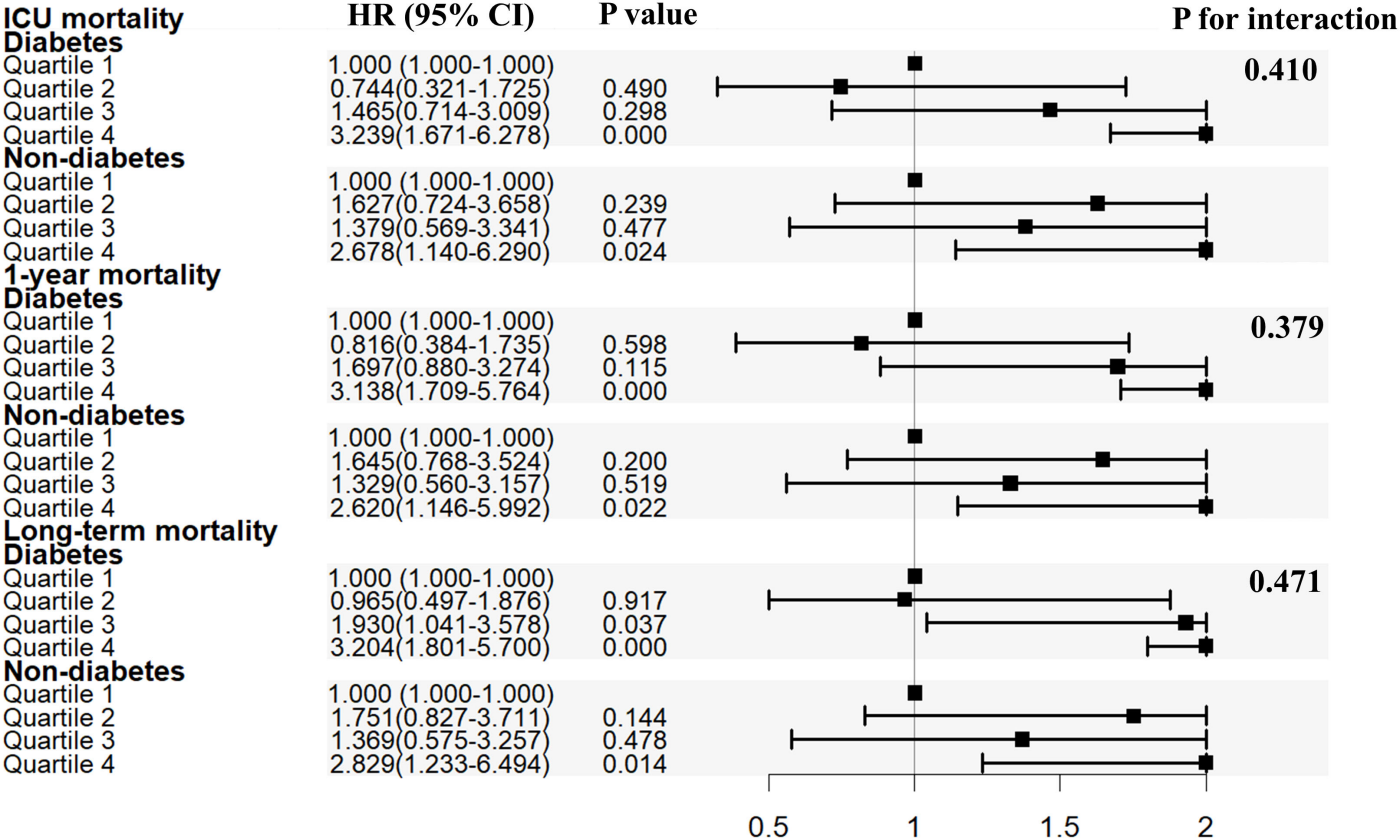
Subgroup analysis for the association of SHR with in ICU mortality, 1-year mortality and overall death. SHR, stress hyperglycemia ratio; ICU, intensive care unit; HR, hazard ratio; CI, confidence interval.

Subgroup analyses were conducted to assess the relationship between SHR and all-cause mortality based on diabetes status (**Table 2**; **Figure 3**). SHR in quartile 4 was significantly associated with an increased risk of ICU mortality in the MIMIC-IV cohort (HR = 3.239, 95% CI: 1.671-6.278, *P* < 0.001; HR = 2.678, 95% CI: 1.140-6.290, *P* = 0.024) compared to SHR in quartile 1 among patients with or without diabetes. Comparable results were found that SHR in quartile 4 was strongly related with higher 1-year mortality (patients with diabetes: HR [95% CI] = 3.138 [1.709-5.764], *P* < 0.001; patients without diabetes: HR [95% CI] = 2.620 [1.146-5.992], *P* = 0.022) and long-term mortality (patients with diabetes: HR [95% CI] = 3.204 [1.801-5.700], *P* < 0.001; patients without diabetes: HR [95% CI] = 2.829 [1.233-6.494], *P* = 0.014). The P value for interaction was 0.410, 0.379, and 0.471 for ICU mortality, 1-year mortality, and long-term mortality, respectively.

**Table 2:** Multivariable Cox regression analysis for ICU mortality, 1-year and long-term all-cause mortality.

	Events (rate, %)	HR (95% CI)	P value
ICU mortality			
SHR (continuous)	128 (23.9)	1.780 (1.506-2.104)	<0.001
SHR (categorical)			
Quartile 1	24 (18.8)	Reference	
Quartile 2	28 (21.9)	1.192 (0.688-2.065)	0.532
Quartile 3	31(24.2)	1.558 (0.902-2.693)	0.112
Quartile 4	45 (35.1)	3.029 (1.802-5.090)	<0.001
1-year mortality			
SHR (continuous)	146 (27.3)	1.767 (1.504-2.076)	<0.001
SHR (categorical)			
Quartile 1	28 (19.2)	Reference	
Quartile 2	33 (22.6)	1.225 (0.738-2.033)	0.432
Quartile 3	35 (24.0)	1.632 (0.979-2.719)	0.060
Quartile 4	50 (34.2)	3.057 (1.885-4.958)	<0.001
Long-term mortality			
SHR (continuous)	169 (31.6)	1.799 (1.536-2.107)	<0.001
SHR (categorical)			
Quartile 1	38 (22.4)	Reference	
Quartile 2	39 (23.1)	1.302 (0.821-2.066)	0.263
Quartile 3	39 (23.1)	1.761 (1.091-2.844)	0.021
Quartile 4	53 (31.4)	3.183 (2.020-5.015)	<0.001

SHR, stress hyperglycemia ratio; ICU, intensive care unit; HR, hazard ratio; CI, confidence interval.

The Multivariable Cox models adjusted for age, gender, hypertension, diabetes mellitus, chronic kidney disease, acute myocardial infarction, cerebrovascular disease, peptic ulcer disease, and chronic obstructive pulmonary disease.

## Discussion

This was the first investigation into the correlation between the SHR, short and long-term all-cause mortality in individuals who have experienced cardiac arrest. Our findings revealed that (1) elevated levels of SHR in cardiac arrest patients admitted to the ICU were associated with increased ICU-mortality, 1-year and long-term all-cause mortality; (2) Increased SHR was significantly correlated with a greater likelihood of all-cause mortality among non-diabetic cardiac arrest patients. This research established SHR as a straightforward yet effective biomarker for assessing risk among ICU-admitted patients who have experienced cardiac arrest.

It was suggested that the hyperactivated oxidative stress response, insulin resistance, inflammation, cytokine production, and hormonal abnormalities may be responsible for the correlation between stress hyperglycemia and unfavorable outcomes (24), despite the fact that the underlying mechanisms are too complicated to fully comprehend. *In vitro* (25) and in patients (26) with or without known diabetes, repetitive acute glucose fluctuations would cause increased endothelial apoptosis, endothelial dysfunction, and oxidative stress responses in comparison to less varied excursions.

It was previously believed that transient hyperglycemia during severe illness in adult patients without known diabetes would be beneficial or even innocuous (27). Initial enthusiasm for strict glycemic control in combined medical and ICU had been tempered by subsequent reports (28–30), primarily due to the unacceptable risk of hypoglycemia. In contrast, the findings of a substantial randomized controlled trial (31) demonstrated unequivocal reductions in mortality associated with intensive insulin therapy for ICU patients, regardless of prior diabetes diagnosis. These discoveries have prompted calls for targeted initiatives to identify patients who are particularly susceptible to damage caused by hyperglycemia and are likely to derive benefits from interventions.

The use of admission glucose as a marker for stress hyperglycemia has been questioned, as a high value does not necessarily indicate an acute increase in glucose in response to severe illnesses, particularly in patients with diabetes and inadequate glycemic control (32). By introducing HbA1c, the SHR, an index of relative glycemia, was developed in an effort to obtain new insights into the relationship between hyperglycemia and patient outcomes. Previous studies had demonstrated that the strong relationship between SHR and poor clinical outcomes in acute coronary syndrome (33–36), acute ischemic stroke (13, 37), heart failure and type 2 DM (38). Our study also demonstrated the strong association between SHR and mortality in cardiac arrest patients.

Higher SHR was associated with a greater risk of all-cause mortality among critically ill patients who have cardiac arrest, and this association is similar to those without diabetes. Previous study also found that hyperglycemia was common in both diabetics and non-diabetics in cardiac arrest patients (39). Diabetes patients had relatively insensitive survival odds to blood glucose, with severe hyperglycemia being the factor associated with decreased survival. Survival probabilities were sensitive to hypoglycemia in non-diabetic individuals (39). A retrospective investigation of critically ill patients failed to establish a significant correlation between average blood glucose concentration and mortality in diabetic patients (40).

ICU-admitted patients frequently exhibit hemodynamic instability, necessitating prompt implementation of optimal care and management. In an effort to enhance patient outcomes, our findings highlight the critical nature of meticulous glycemic control in cardiac arrest patients admitted to intensive care units. Moreover, distinct glycemic management strategies should be implemented for patients with abnormally elevated SHR upon admission, particularly those who do not have diabetes. The mechanisms underlying the relationship between stress hyperglycemia and outcomes in critical cardiac arrest patients require additional research.

## Limitation

We fully recognized a number of limitations in our research. Due to the retrospective nature of this investigation, information regarding potential confounding variables, such as the duration of diabetes, was absent. This real-world study aimed to establish a substantial correlation between increased SHR and all-cause mortality in critically ill cardiac arrest patients, as well as to elaborate on the findings regarding cohorts from the United States.

## Conclusion

The SHR index demonstrates a L-shaped correlation with both ICU, one-year and long-term all-cause mortality in the American MIMIC-IV cohort, according to our research. SHR can serve as a significant prognostic risk indicator for cardiac arrest patients. To investigate the effect of glycemic control according to the SHR on improving outcomes for patients with cardiac arrest, additional prospective studies are required.

## Data availability statement

The raw data supporting the conclusions of this article will be made available by the authors, without undue reservation.

## Ethics statement

The study was performed according to the guidelines of the Helsinki Declaration. The use of the MIMIC-IV database was approved by the review committee of Massachusetts Institute of Technology and Beth Israel Deaconess Medical Center. The data is publicly available (in the MIMIC-IV database), therefore, the ethical approval statement and the requirement for informed consent were waived for this study.

## Author contributions

L-YL: Conceptualization, Data curation, Formal analysis, Writing – original draft. W-HX: Software, Supervision, Validation, Writing – review & editing. J-JL: Conceptualization, Data curation, Writing – review & editing. R-JZ: Conceptualization, Resources, Visualization, Writing – review & editing.

## Supplementary material

Supplementary Figure 1 Restricted cubic spline regression analysis of SHR with in ICU mortality **(A)**, 1-year mortality **(B)** and overall death **(C)**. SHR, stress hyperglycemia ratio; ICU, intensive care unit.
