# Supplementary figures and images for: Impact of stress hyperglycemia ratio on mortality in patients with cardiac arrest: insight from American MIMIC-IV database
Source: Front Endocrinol (Lausanne). 2024 May 21;15:1383993. doi: 10.3389/fendo.2024.1383993 (PMC11148256; doi:10.3389/fendo.2024.1383993)

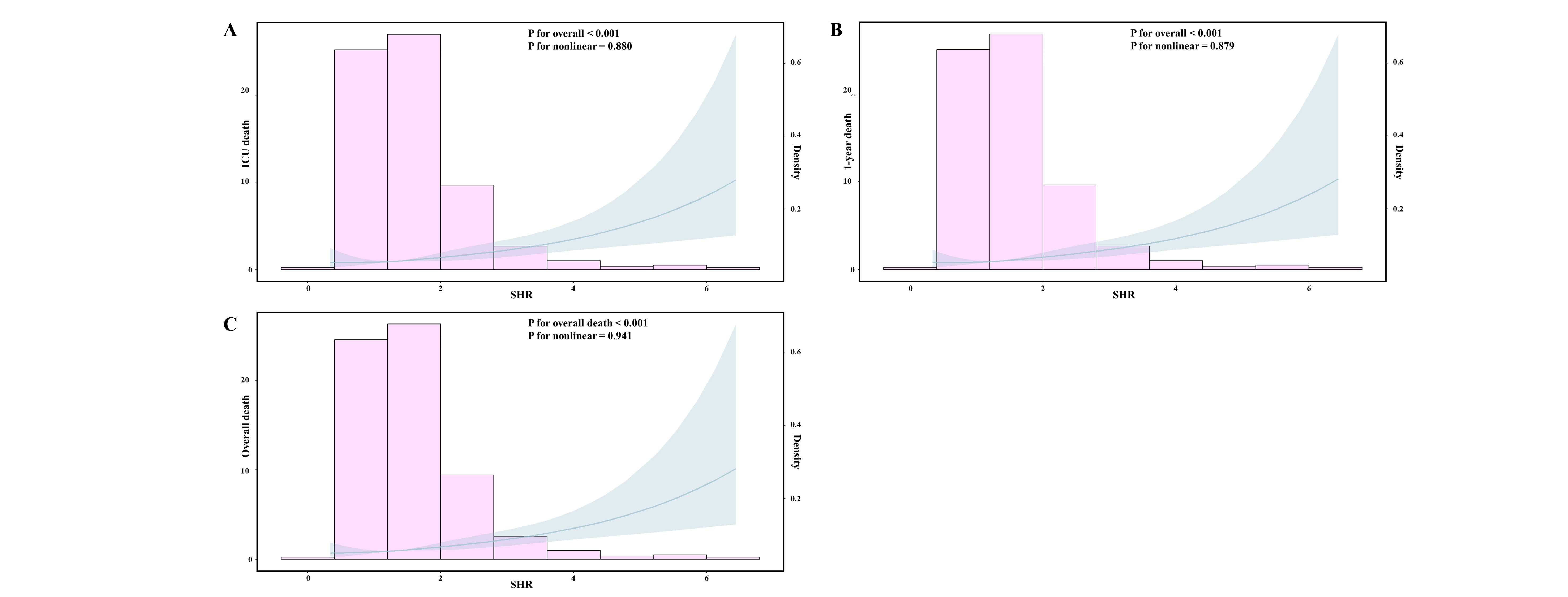

Supplement: Supplementary Figure 1 — Restricted cubic spline regression analysis of SHR with in ICU mortality (A), 1-year mortality (B) and overall death (C). SHR, stress hyperglycemia ratio; ICU, intensive care unit. [file Image_1.tif]
